# Artificial intelligence driven exposome and multi omics integration for biomarker discovery in liver cancer: a literature review

**DOI:** 10.3389/fimmu.2026.1866011

**Published:** 2026-07-23

**Authors:** Xuelian Wang, Yunjing Gao, Ran Xu, Xin Lan, Qiushi Huang

**Affiliations:** 1 Department of Oncology and Hematology, Zhongxian People’s Hospital, Chongqing, China; 2 Department of Pathology, Chongqing General Hospital, Chongqing University, Chongqing, China; 3 School of Clinical Medicine, North Sichuan Medical College, Nanchong, China; 4 Department of Nuclear Medicine, Chongqing General Hospital, Chongqing University, Chongqing, China; 5 Department of Clinical Laboratory, Suining First People’s Hospital, Suining, China

**Keywords:** artificial intelligence, biomarker discovery, exposome, hepatocellular carcinoma, immune microenvironment, liquid biopsy, MASLD-related hepatocellular carcinoma, multi-omics

## Abstract

Primary liver cancer is a major global cause of cancer death, and hepatocellular carcinoma (HCC) is the predominant histological subtype. This literature review synthesizes current evidence on the exposome, multi-omics landscape, and artificial intelligence (AI)-based integration strategies relevant to biomarker discovery in liver cancer, with a focus on biological rationale, emerging clinical applications, and translational limitations. Key etiologic drivers include viral hepatitis, alcohol-related liver disease, and metabolic dysfunction-associated steatotic liver disease, all of which interact with environmental exposures across the life course. Biomarker discovery increasingly relies on integrated assessment of exposure-related signals together with genomic, epigenomic, transcriptomic, proteomic, metabolomic, and spatially resolved data. Hepatocarcinogenesis involves a complex interplay of chronic liver injury, environmentally patterned molecular perturbation, and dynamic tumor-host interactions. We emphasize an exposome-informed, multimodal strategy in which interpretable AI models identify clinically relevant signatures for early detection, prognostic stratification, and treatment guidance. Critical limitations of current evidence include incomplete exposure assessment, heterogeneous data platforms, retrospective study design, limited external validation, and insufficient model transparency. Emerging approaches, including proteogenomic, lipidomic, single-cell, and digital pathology-based modeling, show promise but require further validation in etiologically diverse cohorts. The purpose of this review is to critically examine how AI can integrate exposome-related information with multi-omics data for biomarker discovery in liver cancer. Here, particular attention is given to the exposure-to-biomarker sequence, immune-metabolic remodeling, liquid-biopsy translation, and the reduction of high-dimensional signatures into clinically deployable assays.

## 1 Introduction

Primary liver cancer remains a major global cause of cancer death, and hepatocellular carcinoma (HCC) constitutes the predominant histological subtype (1, 2). Despite progress in surveillance, imaging, locoregional therapy, and systemic treatment, many patients are still diagnosed beyond the stage at which curative intervention is feasible, and no ideal biomarker has yet achieved consistent performance for early detection or precise risk stratification (3, 4). The etiological profile of HCC is also evolving, with increasing contributions from alcohol-related liver disease and metabolic dysfunction-associated steatotic liver disease alongside the persistent burden of viral hepatitis (5, 6). Under these conditions, biomarker discovery must move beyond isolated analytes and toward integrated models that more accurately reflect disease initiation, progression, and interpatient heterogeneity. A broader analytical framework is therefore required to connect causative exposures with molecular alterations and clinically useful classification.

The exposome offers such a perspective by considering the cumulative impact of external and internal exposures across the life course, including pollutants, dietary and lifestyle factors, occupational chemicals, microbial metabolites, and host metabolic responses (7, 8). In liver disease research, exposomic studies have highlighted the relevance of air pollution, volatile compounds, endocrine-disrupting contaminants, per- and polyfluoroalkyl substances, and perturbations of the gut-liver axis, all of which may influence hepatocarcinogenesis through inflammatory, metabolic, and epigenetic mechanisms (9, 10). Human evidence is beginning to support this relationship more directly, as prediagnostic PFOS exposure has been associated with increased non-viral HCC risk together with disturbances in glucose, amino acid, and bile acid metabolism (11, 12). These findings indicate that environmentally patterned molecular change should be regarded as part of the biomarker landscape rather than merely as background etiologic information. This perspective is particularly relevant for liver cancer because the liver is both a target and a processor of many lifelong exposures.

At the same time, HCC is a biologically heterogeneous malignancy whose clinically relevant features cannot be captured adequately by single-omic measurements alone. Multi-omics strategies that integrate genomic, epigenomic, transcriptomic, proteomic, and metabolomic data are increasingly being applied to identify candidate biomarkers, define molecular subtypes, and predict therapeutic response (13, 14). Recent reviews in liver cancer and precision oncology indicate that machine learning and deep learning methods are now central to this effort because they can model nonlinear relationships across disparate data layers and detect complex feature patterns that are difficult to resolve using conventional analytical pipelines (15, 16). However, the translational value of these approaches remains constrained by limited external validation, challenges in data harmonization, restricted cohort diversity, and insufficient interpretability for routine clinical implementation. For liver cancer, the convergence of exposome science, multi-omics profiling, and artificial intelligence is especially important because disease risk and tumor behavior are jointly shaped by chronic liver injury, environmental context, and dynamic tumor-host interactions (17–19). An analytical model that omits exposure history may miss upstream drivers of molecular dysregulation, whereas an exposure-centered approach without deep molecular phenotyping may fail to identify stable and mechanistically informative biomarkers. The purpose of this review is to critically examine how artificial intelligence can integrate exposome-related information with multi-omics data for biomarker discovery in liver cancer, with emphasis on biological rationale, current methodological strategies, emerging clinical applications, and major barriers to translation. This review is intended to clarify the present evidence base and define priorities for future research. The distinctive contribution of this review is therefore not another summary of HCC omics or AI methods alone, but an exposome-to-biomarker framework that connects exposure measurement, immune and metabolic dysregulation, multi-omic perturbation, model-based integration, and clinical assay development.

### 1.1 Review method and literature selection.

For transparency, the literature was identified through targeted searches of PubMed/MEDLINE, Web of Science, Scopus, and Google Scholar from database inception to the final revision, supplemented by citation tracking of major reviews and primary studies. Search terms combined liver cancer, hepatocellular carcinoma, exposome, environmental exposure, PFAS, air pollution, MASLD, multi-omics, genomics, epigenomics, transcriptomics, proteomics, metabolomics, lipidomics, single-cell, spatial omics, artificial intelligence, machine learning, deep learning, liquid biopsy, immune microenvironment, immunotherapy, and biomarker discovery. Priority was given to peer-reviewed human studies, prospective or nested case-control designs, etiologically annotated cohorts, multi-omics or AI-based studies, and clinically oriented biomarker reports. Mechanistic animal or *in vitro* studies were used selectively when they clarified exposure-related immune-metabolic or molecular pathways. Studies were excluded when they lacked liver cancer relevance, did not provide sufficient methodological detail, or made unsupported biomarker claims. Because this article is a narrative literature review rather than a systematic review or meta-analysis, study selection emphasized conceptual relevance, reproducibility, validation strategy, etiologic diversity, and clinical readiness.

## 2 Exposome and liver cancer: conceptual basis for biomarker discovery

The exposome provides a biologically grounded basis for studying liver cancer because it considers the cumulative effect of exogenous and endogenous exposures across the life course. This perspective is especially relevant to hepatocellular carcinoma, since the liver is a principal site of xenobiotic uptake, biotransformation, and inflammatory signaling (20, 21). In practice, hepatocarcinogenesis seldom reflects a single insult. Viral hepatitis, alcohol use, dietary excess, mycotoxins, persistent pollutants, and airborne contaminants act together with host metabolism and chronic tissue injury to shape risk, timing of transformation, and interindividual variability (22, 23). From a biomarker standpoint, useful candidates should therefore not be restricted to indicators of established tumor burden alone. They should also capture exposure history, biological response, and early molecular deviation that precedes radiologically detectable disease.

Conceptually, the exposome-to-biomarker pathway can be organized as a sequential but partly overlapping process: external exposure and exposure mixtures; internal dose, hepatic uptake, and biotransformation; biological response involving oxidative stress, inflammation, immune-metabolic reprogramming, and epigenetic remodeling; preclinical molecular alteration in tissue or circulation; tumor emergence; progression; and response or resistance to therapy. This sequence is useful because it forces each candidate biomarker to be assigned to a biological interval, making it clearer whether the signal reflects risk, early transformation, tumor burden, aggressiveness, or treatment sensitivity (17–23). How exposure is measured is equally important for biomarker discovery. Geospatial air-pollution models and land-use regression can provide long-term external exposure estimates at population scale but may have limited individual specificity; questionnaires and occupational histories capture diet, alcohol, tobacco, work-related agents, and lifestyle patterns but are vulnerable to recall error and coarse timing; plasma or serum chemical measurements and adductomics offer stronger biological specificity for internal dose but may represent recent or episodic exposure unless repeated; metabolomic signatures and untargeted high-resolution mass spectrometry broaden chemical-space coverage but require rigorous annotation, batch control, and cross-platform harmonization (7–12, 17, 24, 25). These differences affect whether an exposure variable is suitable for risk prediction, mechanistic interpretation, or clinical assay development.

Temporal design is critical because many metabolomic, lipidomic, and inflammatory signatures in HCC may be consequences of cirrhosis, tumor burden, cachexia, altered diet, or impaired hepatic clearance rather than causes or early indicators of malignant transformation. Prospective cohorts with prediagnostic biospecimens, repeated sampling, and nested case-control analyses can reduce reverse causation, assess within-person trajectories, and identify signals that appear before imaging-based diagnosis or clinical decompensation (26–30). Direct human and molecular data support this position. Aflatoxin-associated HCC remains a clear example of how a defined exposure can leave tumor-specific molecular features, including characteristic mutational patterns that distinguish an etiologic subset of disease (31, 32). Similarly, prediagnostic PFOS exposure has been linked to increased non-viral HCC risk together with changes in glucose, amino acid, and bile acid metabolism, indicating that exposure-related metabolic disturbance may be measurable before clinical diagnosis (33, 34). Evidence linking ambient air pollution with liver cancer risk further suggests that long-term environmental burden should not be treated as background noise in biomarker studies (35, 36). As shown in **Table 1**, the conceptual value of the exposome lies in connecting external exposure, internal dose, molecular perturbation, and clinical application within the same disease process. The central implication is that biomarker discovery in liver cancer should trace the sequence from exposure to biological effect rather than isolate the tumor from its etiologic context.

**Table 1:** Exposure-to-biomarker sequence and exposome-related dimensions relevant to biomarker discovery in liver cancer.

Domain	What it captures	Relevance to biomarker discovery in liver cancer
External exposure profile	Chemical, dietary, lifestyle, occupational, infectious, and environmental inputs over time	Identifies etiologic burden and exposure mixtures associated with increased risk
Internal dose and biotransformation	Absorbed compounds, metabolites, adducts, and products of hepatic processing	Reflects what has biologically reached the liver and undergone metabolic conversion
Molecular response	DNA methylation change, somatic mutation patterns, transcript shifts, protein change, and metabolomic disturbance	Reveals exposure-related biological effects that may precede overt malignancy
Disease transition	Progressive movement from chronic liver injury to cirrhosis, dysplasia, and HCC	Supports risk stratification, early detection, and etiologic subclassification
Exposure measurement and temporal scale	Geospatial air-pollution models, questionnaires, occupational histories, plasma/serum chemicals, adducts, untargeted high-resolution mass spectrometry, and metabolomic signatures	Determines temporal resolution, measurement error, biological specificity, and feasibility for longitudinal biomarker studies
Preclinical molecular alteration	Exposure-imprinted methylation, immune-metabolic, lipidomic, and inflammatory changes before diagnosis	Helps distinguish early-response biomarkers from late effects of cirrhosis, tumor burden, cachexia, altered diet, or impaired liver function
Immune-metabolic remodeling	Macrophage polarization, T-cell exhaustion, immune exclusion, gut-liver axis signaling, and checkpoint-response context	Links exposure and chronic liver injury to immune biomarker discovery and immunotherapy stratification
Treatment response and resistance	Therapy-associated molecular adaptation, immune escape, and host-response signatures	Extends the exposure-to-biomarker sequence from risk and early detection to clinically actionable treatment guidance

This reasoning is strengthened by epigenetic and liquid-biopsy studies. Distinct DNA methylation patterns in HCC have been associated with etiologic backgrounds such as HBV, HCV, and alcohol exposure (37). More recent reviews of HCC epigenetics indicate that chromatin and methylation alterations are not incidental findings, but stable components of tumor biology with diagnostic and prognostic relevance (38). In parallel, circulating methylated DNA markers have shown promise for blood-based detection of HCC, and their performance appears to depend partly on the underlying cause of liver disease (39). These observations are important for exposome research because they show that prior exposure can be biologically embedded in molecular signals that remain measurable in tissue or circulation. For liver cancer, this provides a mechanistic basis for selecting biomarkers that are informative not only for detection, but also for etiologic interpretation. The contribution of the exposome to liver cancer biomarker discovery is not simply that it broadens the list of candidate exposures. Its greater value is that it organizes biomarker research along the natural history of disease, beginning with chronic exposure, proceeding through metabolic and epigenetic response, and culminating in malignant transformation. This is particularly important in HCC, where several etiologies converge on shared clinical endpoints but may do so through partly distinct molecular routes. An exposome-informed approach to biomarker research therefore favors longitudinal sampling, prediagnostic biospecimens, and etiologically diverse cohorts, because these study features improve the likelihood of identifying biomarkers that remain interpretable across heterogeneous patient populations.

## 3 Multi-omics landscape of liver cancer

**Figure 1:**
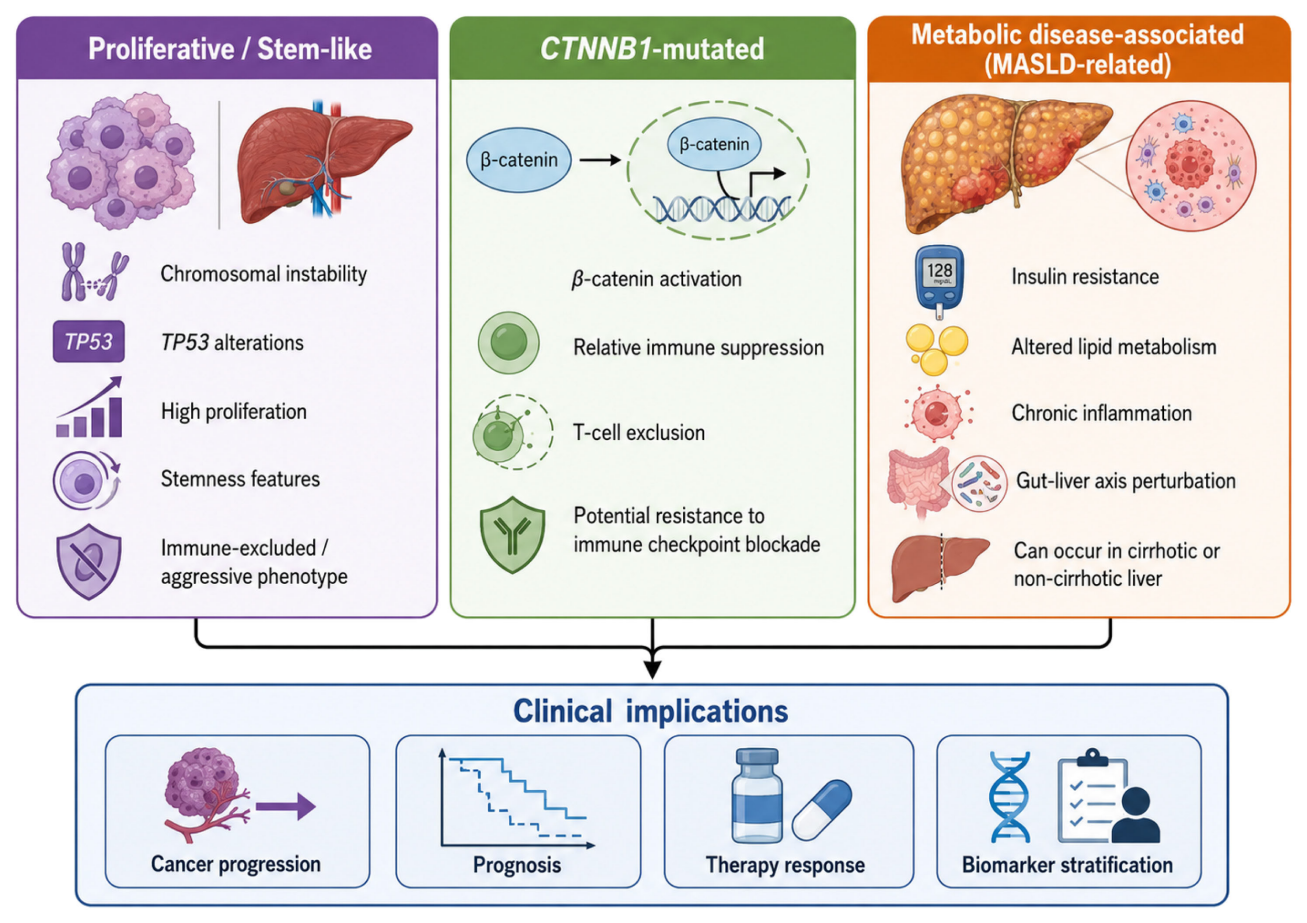
Major molecular and etiologic subclasses of hepatocellular carcinoma and their clinical implications.

The multi-omics landscape of liver cancer shows that hepatocellular carcinoma is not a single molecular entity, but a biologically heterogeneous disease defined by recurrent driver alterations, copy-number change, epigenetic instability, and variable immune and metabolic reprogramming. Large integrative genomic studies identified frequent alterations involving the TERT promoter, TP53, and CTNNB1, together with less common events affecting chromatin regulation, oxidative stress response, and cell-cycle control, whereas more recent whole-genome analyses have further expanded the catalogue of coding and non-coding drivers, mutational signatures, extrachromosomal circular DNA, and subclonal catastrophic events (40, 41). As shown in **Figure 1**, integrative classification studies subsequently defined major subclasses characterized by proliferative or stem-like features with chromosomal instability, CTNNB1-mutated tumors with relative immune suppression, and metabolic disease-associated tumors with distinct inflammatory composition and clinical behavior (42). These findings indicate that genomic information alone does not fully account for the biological diversity relevant to biomarker discovery.

**Figure 2:**
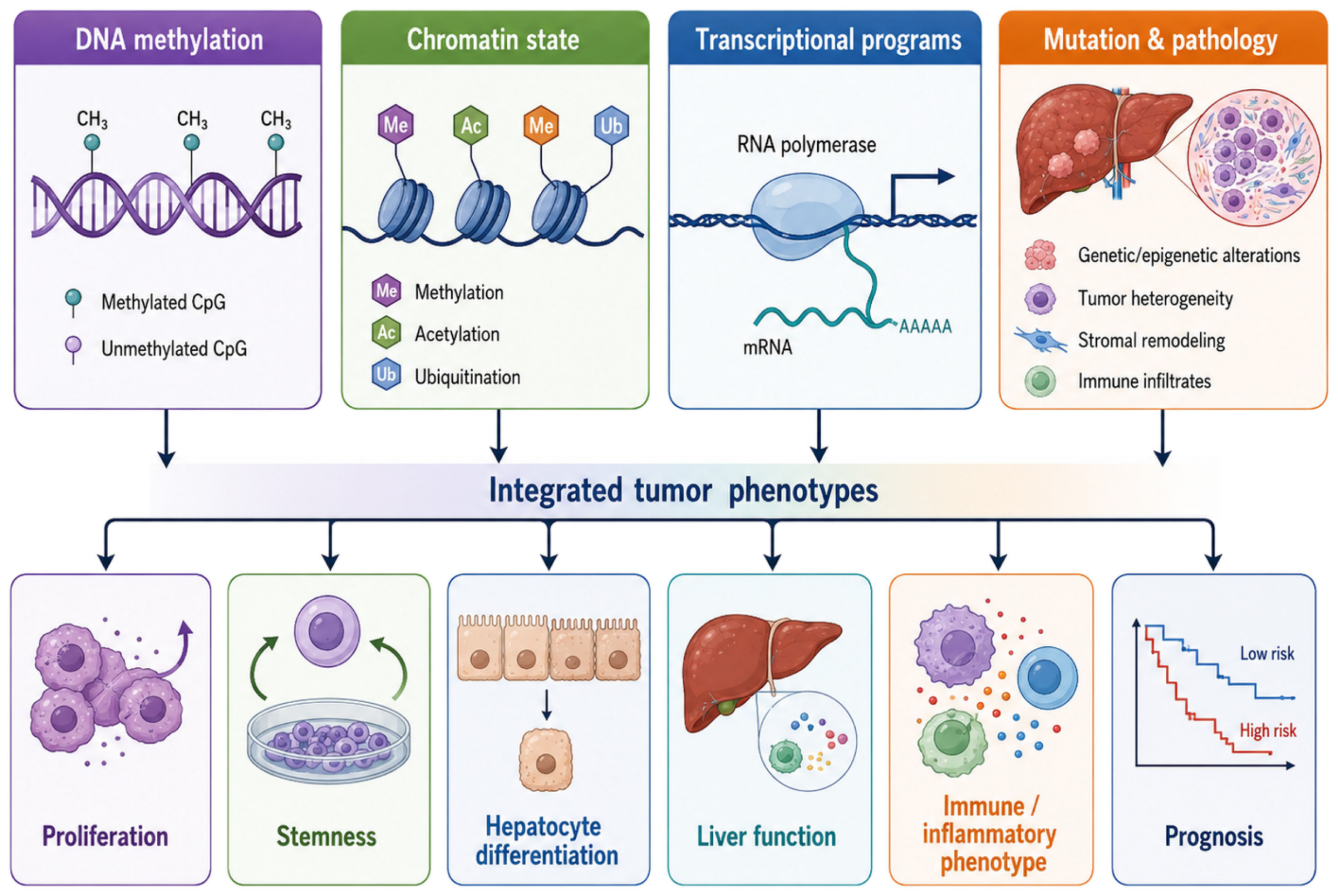
Epigenomic and transcriptomic determinants of tumor phenotypes in hepatocellular carcinoma.

As shown in **Figure 2**, at the epigenomic and transcriptomic levels, liver cancer exhibits coordinated variation in DNA methylation, chromatin state, and lineage-associated transcriptional programs that are closely linked to tumor phenotype. Datasets integrating gene expression, methylation, mutation status, and clinicopathological annotation have shown that transcriptomic subclasses differ in proliferation, stemness, hepatocyte differentiation, liver function, and prognosis, while histological subtypes are strongly associated with specific gene alterations and molecular classes (43, 44). Epigenetic studies further indicate that aberrant DNA methylation, histone modification, and chromatin-remodeling events participate from preneoplastic stages onward and contribute to dedifferentiation, metabolic dysregulation, and therapeutic resistance (45). For this reason, transcript-level candidates are unlikely to be interpreted correctly unless their epigenetic context is considered at the same time.

Proteomic and phosphoproteomic studies have materially refined the interpretation of liver cancer heterogeneity because messenger RNA abundance and protein activity are not interchangeable measures. In early-stage HBV-related hepatocellular carcinoma, proteomic profiling identified three clinically distinct subtypes, and the subgroup characterized by disturbed cholesterol homeostasis and high SOAT1 expression showed particularly adverse outcome together with experimental sensitivity to SOAT1 inhibition (46, 47). Subsequent HBV-related and etiology-diverse proteogenomic analyses demonstrated both concordance and discordance across the genome, transcriptome, proteome, and phosphoproteome, and linked specific molecular clusters to metabolic reprogramming, microenvironmental dysregulation, kinase activation, and altered Wnt/β-catenin, AKT/mTOR, Notch, cell-cycle, and DNA-damage signaling (48, 49). Proteogenomic data therefore provide not only candidate biomarkers, but also functional information that helps determine which abnormalities are most likely to be clinically informative.

Single-cell analyses have shown that liver cancer is organized by interactions among malignant hepatocytes, myeloid populations, lymphocyte subsets, endothelial cells, and stromal cells rather than by tumor-cell features alone. High-resolution immune profiling demonstrated marked diversity among exhausted T cells, regulatory T cells, dendritic cells, and macrophage states in hepatocellular carcinoma, and later atlases of primary and metastatic disease identified heterogeneous malignant hepatocyte programs, terminal MMP9-positive tumor-associated macrophages, and microenvironment-based subtypes associated with prognosis (50, 51). Multiregional and multidimensional single-cell studies further showed that interregional variation within a tumor may be smaller than interpatient variation for key cellular programs, identified stable ligand-receptor interactions such as LGALS9-SLC1A5 and SPP1-PTGER4 in aggressive disease, described a suppressive immune milieu in AFP-positive tumors enriched for SPP1-positive macrophage signaling, and demonstrated that distinct oncogenic driver combinations can impose specific myeloid-centered and T-cell-excluded microenvironments (52, 53). These results show that cellular composition and cell-cell signaling are themselves measurable components of biomarker biology.

An immunological interpretation is particularly important because many exposures linked to HCC converge on chronic inflammatory and immune-metabolic pathways. Viral persistence, alcohol exposure, obesity-associated insulin resistance, pollutants, and gut-liver axis perturbation can influence Kupffer-cell activation, macrophage polarization, dendritic-cell maturation, regulatory T-cell accumulation, cytotoxic T-cell exhaustion, and immune exclusion. These processes are reflected across transcriptomic, proteomic, metabolomic, single-cell, and spatial data and may help explain why tumors with similar stage differ in response to immune checkpoint blockade. Therefore, immune biomarkers should be interpreted with the etiologic and exposomic context that shaped the tumor microenvironment (17–19, 50–57). Metabolomic and lipidomic studies add a particularly important layer in liver cancer because tumor development occurs within an organ that governs systemic nutrient handling and xenobiotic biotransformation. Prospective serum metabolomics in cirrhotic cohorts has associated future hepatocellular carcinoma development with alterations in N-acetylglycine, amino acids, bile acids, choline-related metabolites, and microbiota-derived products, while serum lipidomics has shown that NAFLD-associated hepatocellular carcinoma has a distinct circulating lipid profile with diagnostic potential that exceeded AFP in the reported cohort (26, 27). Comparative proteomic analysis across primary liver cancer types also suggests that abnormal lipid metabolism is a defining feature of hepatocellular carcinoma, whereas extracellular-matrix-related programs are more prominent in intrahepatic cholangiocarcinoma, and a small protein panel distinguished these entities with high accuracy (58, 59). This metabolic layer is especially relevant for biomarker work because it connects etiologic background, tumor phenotype, and accessible circulating signals. The multi-omics landscape of liver cancer is best understood as a set of linked but non-equivalent molecular layers in which driver mutations, epigenetic state, transcriptional output, protein activity, metabolite composition, and multicellular organization each contribute partly distinct information. For biomarker discovery, the central implication is that markers derived from a single layer may misclassify biologically divergent tumors that share the same clinical stage, whereas convergent signals observed across multiple layers are more likely to remain robust across etiologies and disease states. A useful strategy should therefore prioritize matched multi-omic sampling, precise etiologic annotation, and validation in both tissue and circulation. Etiologic heterogeneity has direct biomarker implications, and MASLD-related HCC requires particular attention. Compared with viral-associated disease, MASLD-related HCC may occur in non-cirrhotic livers, is closely linked to insulin resistance, adipose-driven inflammation, altered lipid handling, lipotoxicity, bile acid disturbance, and gut-liver axis signaling, and is often detected less efficiently because surveillance performance is reduced in obese patients and routine surveillance is not consistently applied in non-cirrhotic populations (5, 6, 26–28, 30). Biomarkers for this subgroup therefore need to distinguish metabolic liver injury from early malignant transformation, and lipidomic or inflammatory signals should be validated against MASLD controls with and without cirrhosis.

## 4 Artificial intelligence for exposome and multi-omics integration

Artificial intelligence is increasingly required when exposome variables and multi-omic measurements are analyzed together, because these data differ substantially in scale, sparsity, temporal structure, and biological meaning (60, 61). Current integration strategies include feature-level fusion, latent-space integration, model-level fusion, and hierarchy-aware approaches that preserve regulatory relationships among molecular layers (62). In liver cancer, this distinction is especially important because environmental exposures are captured as mixtures, internal dose markers, or exposure-imprinted metabolites, whereas tumor-associated signals are measured through methylation, RNA, protein, phosphoprotein, glycan, or lipid readouts (63). Under these conditions, AI is useful not simply because it improves prediction, but because it can recover non-linear cross-modal associations that are difficult to detect when each data layer is analyzed in isolation. The central objective is therefore the identification of biomarker combinations that remain biologically interpretable across distinct etiologic backgrounds.

A unified AI framework should therefore begin with exposure measurement, pass through chronic liver injury and immune-metabolic dysregulation, align these features with matched molecular layers, and only then test whether the resulting signatures improve decision-making for surveillance, diagnosis, prognosis, or therapy selection. In this structure, AI is not a substitute for biological design; it is the integration layer that links temporality, etiology, and molecular state. As shown in **Table 2**, the most informative use of AI in this setting is to connect exposure characterization, cross-omic feature selection, subtype definition, and clinically relevant risk estimation within a single analytic process. Direct liver-cancer studies that jointly quantify the external exposome, internal chemical burden, and matched tumor multi-omics remain limited. However, the available evidence already shows why such integration is necessary (64, 65). In a prediagnostic human study, high PFOS exposure was associated with increased non-viral HCC risk together with perturbation of glucose, amino acid, glycan, and bile acid pathways (66). In parallel, liver-focused exposome analyses indicate that air pollutants, persistent contaminants, and gut-liver metabolic signals leave measurable molecular effects that can be followed through metabolomic and epigenomic data layers (67, 68). These finding support the view that exposure-derived variables should be treated as mechanistically relevant inputs for biomarker discovery rather than as background covariates.

**Table 2:** Core analytical and translational roles of artificial intelligence in exposome and multi-omics integration for biomarker discovery in liver cancer.

Analytic component	Typical inputs	Main contribution to biomarker discovery
Exposure characterization	Environmental mixtures, lifestyle factors, occupational agents, infectious history, internal dose markers	Identifies exposure patterns and exposure-related internal signatures relevant to hepatocarcinogenesis
Cross-omic alignment	DNA methylation, transcriptomics, proteomics, phosphoproteomics, metabolomics, glycomics, lipidomics	Selects coordinated features that connect exposure response with tumor biology
Subtype and trajectory modeling	Matched molecular profiles, single-cell data, spatial data, longitudinal samples	Defines biologically coherent subgroups and disease-transition states
Prediction and interpretation	Integrated exposure and omics variables linked to diagnosis, prognosis, or treatment response	Produces clinically usable biomarker panels and clarifies dominant contributors to model output
Critical appraisal and validation	Sample size, cohort diversity, missing data patterns, external cohorts, calibration, and clinical decision endpoints	Separates biologically plausible discovery signals from reproducible biomarkers that are ready for clinical testing
Feature reduction and assay conversion	Model-selected proteins, methylation markers, metabolites/lipids, immunohistochemical markers, and clinical variables	Converts high-dimensional signatures into targeted proteomic panels, PCR assays, metabolite panels, immunohistochemistry, or simplified risk scores

This analytic direction is increasingly supported by HCC studies that integrate several molecular layers with AI-based modeling. Xing et al. combined genomic, transcriptomic, proteomic, and phosphoproteomic profiles from 160 tumors and identified three proteomic subtypes with distinct prognosis, pathway activity, and predicted sorafenib sensitivity; a simplified nine-protein panel was then proposed for potential clinical use (69). Using spatial glycan imaging with glycoproteomics, Wang et al. generated machine-learning models that distinguished early HCC and cirrhosis with AUROC values of 0.88-0.97, indicating that cross-platform integration can yield blood-accessible early-detection markers (70). More recent studies extended this approach to prognosis and microenvironmental stratification. A multi-center analysis across six HCC cohorts comprising 1,110 cases derived a seven-gene consensus AI signature that outperformed conventional clinical variables and 150 published signatures (71), while another multi-omics study integrated mRNA, lncRNA, miRNA, DNA methylation, mutation, single-cell, and spatial data to define four molecular subtypes and a five-gene risk model linked to immune evasion and regional tumor heterogeneity (54). Explainable deep learning has also been applied to identify 12 lipid biomarkers spanning the inflammatory-to-cancer transition (72, 73), and plasma proteomics combined with machine learning has produced a three-protein model for preoperative microvascular invasion risk stratification. The current study remains constrained by several persistent limitations. Many models are trained on retrospective public cohorts with incomplete exposure annotation, and external exposome information is often inferred indirectly from metabolomic disturbance rather than measured prospectively. Missing data across omic layers, cohort imbalance, and limited model transparency further restrict clinical generalizability. For liver cancer, the next step is to design studies in which longitudinal exposure assessment, prediagnostic biospecimens, matched tissue and circulating analytes, and interpretable multimodal learning are evaluated within the same cohort.

Critical appraisal of this literature shows a clear gradient of readiness. Studies with matched multi-omic layers, independent validation, etiologic annotation, and clinically meaningful endpoints offer the strongest evidence, whereas models derived from small retrospective public cohorts, single-center samples, or highly selected datasets remain mainly hypothesis-generating. The reproducibility of reported signatures depends on feature stability, preprocessing choices, platform batch effects, class imbalance, missing omic layers, and whether validation is truly external (29, 74–77). For this reason, AUROC or concordance alone should not be viewed as sufficient for clinical readiness unless accompanied by calibration, decision-curve assessment, assay feasibility, and comparison with current surveillance or staging tools.

## 5 AI-driven biomarker discovery in liver cancer

Artificial intelligence has expanded biomarker discovery in liver cancer from single-analyte screening to the identification of diagnostic, prognostic, and treatment-response signatures that reflect coordinated biological states. In hepatocellular carcinoma, this is particularly important because molecular heterogeneity often exceeds what can be resolved by conventional clinicopathological classification alone. Integrative proteogenomic work in HBV-related tumors showed that cross-platform analysis can define survival-associated subgroups and prioritize biomarkers linked to metabolic rewiring rather than to isolated expression change, including PYCR2 and ADH1A (48, 78). More recently, machine learning applied to multi-omics datasets together with single-cell and spatial transcriptomic information identified four molecular subtypes and a five-gene prognostic model, with the low-risk group showing a more inflamed tumor phenotype and greater predicted benefit from immunotherapy (54, 55). These findings indicate that AI contributes most when it captures convergent molecular organization across data layers instead of ranking candidates within a single omic matrix.

Circulating biomarker discovery has advanced in parallel, especially in etiologically defined disease states where exposure-related metabolic disturbance is biologically relevant. In non-alcoholic fatty liver disease-associated HCC, machine learning analysis of serum lipidomics identified a diagnostic score based on a marked reorganization of the circulating lipidome, and this model showed stronger diagnostic discrimination than AFP while separating metabolic-disease-associated tumors from viral- and alcohol-related HCC (27, 28). A distinct blood-based strategy used data-independent plasma proteomics and machine learning to generate the PRIM model for preoperative prediction of microvascular invasion from TALDO1, PDIA3, and PGK1, with additional single-cell analysis showing that these markers were concentrated mainly in malignant cells and macrophages within the invasive tumor microenvironment (73, 79). For an exposome-oriented framework, such studies are especially valuable because they preserve information on chronic metabolic injury, host response, and aggressive tumor behavior within clinically accessible biospecimens.

Liquid biopsy deserves particular emphasis because it offers a practical route for repeated sampling across the long transition from chronic liver disease to cancer. Circulating methylated DNA, cell-free DNA features, proteins, glycoproteins, metabolites, lipids, and immune mediators can capture tumor-derived signals together with host response and exposure-imprinted biology, making them suitable for surveillance and early-detection settings where tissue is unavailable (27, 28, 39, 70, 72, 73, 79). However, these assays must be calibrated against cirrhosis severity, liver function, hemolysis, diet, obesity, renal function, and inflammatory activity, because these factors can dilute or mimic tumor-related signals. Paired tissue-plasma studies and longitudinal prediagnostic sampling are therefore necessary before liquid-biopsy markers can be considered clinically actionable.

AI is also converting routine histology into a source of molecularly informative biomarkers. Zeng et al. showed that deep-learning models trained on digitized hematoxylin-eosin slides could predict six immune and inflammatory gene signatures in HCC, with validation AUCs ranging from 0.81 to 0.92, thereby demonstrating that tissue morphology can function as a surrogate for transcriptomic immune states (56). Seraphin et al. extended this concept by showing that transformer-based pathology models could identify poor-prognosis molecular subclasses and microvascular invasion from a single H&E slide, and that AI-predicted vascular invasion was associated with shorter survival and an immunosuppressive microenvironment (57). This strategy is attractive for biomarker translation because it reduces dependence on large molecular platforms while retaining biologically relevant stratification.

Imaging-based AI biomarkers extend the same principle into treatment stratification. In patients with unresectable HCC receiving atezolizumab plus bevacizumab, an integrated radiomic-clinical model derived from pretreatment CT outperformed conventional indicators such as BCLC stage and ALBI grade for prediction of 12-month mortality, and it separated groups with clearly different overall and progression-free survival (80, 81). Current evidence suggests that the most useful biomarkers in liver cancer will arise not from isolated platforms, but from AI systems capable of jointly interpreting etiologic context, circulating analytes, tissue architecture, and molecular programs. Clinical translation will therefore depend on prospective and etiologically diverse cohorts in which predictive performance, interpretability, and practical clinical value are evaluated at the same time.

## 6 Current challenges and limitations

Despite the growing number of studies in this area, true integration of exposome assessment with multi-omics and artificial intelligence in liver cancer remains uncommon. A recent systematic review of multi-omics research in liver cancer concluded that clinical validation is still limited and that major unresolved problems include data integration, model interpretability, and inadequate cohort diversity (29). In parallel, exposomic analysis continues to face a narrower but equally important limitation: current analytical workflows do not yet capture the full internal chemical exposome with sufficient sensitivity, annotation depth, and inter-study harmonization, while exposome-wide association studies must also accommodate temporal variability, correlated mixtures, and non-uniform exposure measurement (24, 25). Consequently, many candidate biomarkers are linked to only partially measured exposure histories, and the biological sequence connecting chronic exposure to molecular change and tumor emergence is often incompletely defined.

Limitations in dataset structure further restrict robust biomarker discovery. Omics-derived biomarkers in liver disease remain affected by semi-quantitative measurements, substantial analytical variation, and the absence of standardized validation across populations (30). More broadly, data-driven omics integration is challenged by high dimensionality, collinearity, missing values, mismatched data types, and batch effects, and these problems become more pronounced when metabolomic, proteomic, epigenomic, transcriptomic, clinical, and exposure variables are analyzed together (82, 83). In addition, a recent oncology review found that many AI-based multi-omics studies still rely predominantly on public cancer resources and remain at proof-of-concept stage rather than real-world deployment (74). Under these conditions, an apparently stable signature may reflect dataset-specific structure rather than a reproducible liver-cancer biomarker.

Model transparency and transportability also remain major barriers to clinical use. Current hepatocellular carcinoma pathomics literature identifies data heterogeneity, limited interpretability, ethical and regulatory uncertainty, and the absence of standardized protocols as central reasons why promising AI systems rarely progress into routine care (75). Although recent multicenter imaging studies have shown that interpretable and externally tested models are achievable in hepatocellular carcinoma, including MRI-based microvascular invasion prediction and CT-based early-warning models in cirrhotic cohorts (76, 77), such designs are still exceptional and are usually developed without simultaneously measured lifetime exposure data, repeated biospecimens, and matched tissue-circulation multi-omics. The result is a persistent gap between predictive performance under study conditions and reliability across institutions, laboratories, and patient populations.

Another major limitation is the shortage of longitudinal, etiologically diverse cohorts in which exposure assessment and multi-layer molecular profiling are collected over time. Recent methodological work has emphasized that AI-based biomedical discovery depends heavily on longitudinal data integrating omics with imaging, clinical variables, diet, drugs, toxins, and other environmental information (84). For liver cancer, this requirement is especially important because biomarker trajectories differ across chronic viral hepatitis, alcohol-related injury, and metabolic liver disease, yet most available datasets remain retrospective and insufficiently synchronized across these domains. For this reason, the present evidence base is more suitable for generating biologically plausible candidates than for establishing clinically qualified biomarkers ready for routine implementation.

## 7 Future perspectives and clinical translation

Clinical translation in this field will depend on moving beyond retrospective association studies toward longitudinal designs in which exposure assessment, serial blood sampling, imaging, and matched tissue profiling are collected within the same patients. This requirement is particularly important in liver cancer because exposure-related metabolic and epigenetic alterations may precede radiological disease and may differ substantially across viral hepatitis, alcohol-related liver disease, and metabolic dysfunction-associated steatotic liver disease. Cohorts that include prediagnostic biospecimens and repeated follow-up will be better positioned to distinguish biomarkers of early malignant transformation from markers of established tumor burden, while also allowing direct evaluation of temporal stability, lead time, and etiologic specificity.

At the analytical level, clinically useful models must integrate exposomic variables with molecular, imaging, and pathological data while remaining interpretable, externally valid, and operationally feasible. Current studies have shown that multi-omics artificial intelligence models can identify prognostic subtypes, lipid-defined transition states, and protein signatures with strong discriminatory performance, but these systems remain affected by batch effects, missing data, cohort imbalance, and limited transportability across institutions (29, 77). The immediate priority is therefore not additional model complexity alone, but standardized preprocessing, rigorous external validation, calibration across etiologic strata, and reduction of large feature sets into analytically robust panels that can be measured reproducibly in clinical laboratories.

Translation also requires explicit reduction of discovery-stage features into deployable assays. High-dimensional multi-omics signatures should be narrowed through stability selection, penalized modeling, biological pathway grouping, and independent validation, then converted into targeted proteomic panels, methylation PCR or digital PCR assays, metabolite or lipid panels, immunohistochemistry, or simplified risk scores. A biomarker panel is clinically credible only if it has defined thresholds, analytic reproducibility, acceptable cost and turnaround time, and incremental value over AFP, imaging, staging systems, and routine liver-function assessment (27–29, 54, 69–73, 79). For clinical adoption, biomarker development should be directed toward clearly defined decision points, including surveillance in high-risk chronic liver disease, distinction of early hepatocellular carcinoma from cirrhosis or dysplastic change, estimation of recurrence risk after curative therapy, and prediction of systemic treatment response. Multimodal signatures that combine circulating proteins, lipids, methylation markers, radiomics, and digital pathology are especially promising because they preserve complementary information on exposure history, tumor biology, and host microenvironment (54, 81). However, translation will require conversion of these signatures into parsimonious assays with clear thresholds, acceptable cost, short turnaround time, and performance that remains superior to conventional markers and routine clinicoradiological assessment in prospective real-world populations.

A further requirement is cohort diversity and reporting transparency. Models developed in narrowly defined populations are unlikely to support broad clinical use in a disease whose incidence, exposure profile, and molecular determinants vary across regions and etiologies (29, 84). Future studies should therefore incorporate multinational recruitment, standardized exposure annotation, matched tissue and liquid-biopsy sampling, and transparent reporting of model behavior. Under these conditions, artificial intelligence-driven integration of the exposome and multi-omics may progress from exploratory biomarker discovery to clinically reliable risk stratification and treatment guidance, with the greatest value likely to come from biologically interpretable signatures that remain stable across etiologies and across the continuum of liver carcinogenesis.

## 8 Conclusion

Artificial intelligence-driven integration of exposome data with multi-omics profiling represents a promising direction for biomarker discovery in liver cancer because it addresses the combined influence of environmental exposure, chronic liver injury, and molecular heterogeneity on hepatocarcinogenesis. Current evidence indicates that exposome-informed analyses can reveal biologically relevant metabolic and epigenetic perturbations linked to liver cancer risk, while multi-omics approaches provide complementary information on genomic, transcriptomic, proteomic, and metabolomic variation that cannot be resolved adequately by single-layer studies alone. In this setting, artificial intelligence is most valuable when it identifies coordinated signals across these domains and translates them into candidate biomarkers for early detection, prognostic stratification, and treatment guidance. At the same time, clinical application remains limited by incomplete exposure assessment, heterogeneous platforms, retrospective study design, insufficient external validation, and restricted model interpretability across diverse etiological populations. Progress in this field will depend on prospective multicenter studies that combine standardized exposure characterization, serial biospecimen collection, matched tissue and liquid-biopsy profiling, and interpretable analytic models that can be reduced to robust assays for surveillance, early diagnosis, and risk stratification in routine liver cancer care. Concrete priorities are prospective multiethnic and etiologically stratified cohorts; standardized exposure assessment using both external and internal measurements; longitudinal prediagnostic biospecimen collection; matched tissue, single-cell, spatial, and liquid-biopsy profiling; harmonized preprocessing and transparent reporting; externally validated and interpretable AI models; and conversion of complex signatures into scalable assays for surveillance, early diagnosis, recurrence prediction, and immunotherapy-response stratification.
